# SUCCESSFUL AGING A SELF-RELIANT UMBRELLA: DEFINING SUCCESSFUL AGING AMONG THE OLD-OLD (80+) IN SHANGHAI

**DOI:** 10.1093/geroni/igz038.3056

**Published:** 2019-11-08

**Authors:** Lin Chen, Minzhi Ye, Eva Kahana

**Affiliations:** 1 Fudan University, Shanghai, China; 2 Benjamin Rose Institute on Aging, Cleveland, Ohio, United States; 3 Case Western Reserve University, Cleveland, Ohio, United States

## Abstract

The Chinese old-old (80+) population has steadily increased in recent years; however, limited studies have examined how they age. The purpose of this study is to explore how the old-old in urban China define successful aging. Guided by grounded theory, community-dwelling old-old individuals participated in semi-structured, in-depth interviews (N= 97). Participants identified self-reliance as the goal of successful aging, which was supported by four proactive behaviors, including physical activity, financial security, community connectedness, and willing acceptance of reality. These four proactive behaviors were conceptualized to constitute the ribs of an umbrella model offering a canopy to protect the pole of self-reliant successful aging. This study offers new insight in understanding dynamic and nuanced ways that the old-old in urban China age successfully and their valiant efforts to maintain an ideal status.

